# Application of Artificial Intelligence in Shared Decision Making: Scoping Review

**DOI:** 10.2196/36199

**Published:** 2022-08-09

**Authors:** Samira Abbasgholizadeh Rahimi, Michelle Cwintal, Yuhui Huang, Pooria Ghadiri, Roland Grad, Dan Poenaru, Genevieve Gore, Hervé Tchala Vignon Zomahoun, France Légaré, Pierre Pluye

**Affiliations:** 1 Department of Family Medicine McGill University Montreal, QC Canada; 2 Lady Davis Institute for Medical Research Jewish General Hospital Montreal, QC Canada; 3 Mila-Quebec AI Institute Montreal, QC Canada; 4 Faculty of Dental Medicine and Oral Health Sciences McGill University Montreal, QC Canada; 5 Department of Integrated Studies in Education McGill University Montreal, QC Canada; 6 Department of Pediatric Surgery McGill University Health Centre Montreal, QC Canada; 7 Schulich Library of Physical Sciences, Life Sciences, and Engineering McGill University Montreal, QC Canada; 8 Centre de recherche en santé durable, Centre intégré universitaire de santé et services sociaux de la Capitale-Nationale Quebec City, QC Canada; 9 Quebec Support for People and Patient-Oriented Research and Trials Unit Quebec City, QC Canada; 10 Department of Family Medicine and Emergency Medicine Faculty of Medicine Université Laval Quebec City, QC Canada

**Keywords:** artificial intelligence, machine learning, shared decision making, patient-centered care, scoping review

## Abstract

**Background:**

Artificial intelligence (AI) has shown promising results in various fields of medicine. It has the potential to facilitate shared decision making (SDM). However, there is no comprehensive mapping of how AI may be used for SDM.

**Objective:**

We aimed to identify and evaluate published studies that have tested or implemented AI to facilitate SDM.

**Methods:**

We performed a scoping review informed by the methodological framework proposed by Levac et al, modifications to the original Arksey and O'Malley framework of a scoping review, and the Joanna Briggs Institute scoping review framework. We reported our results based on the PRISMA-ScR (Preferred Reporting Items for Systematic Reviews and Meta-Analyses extension for Scoping Reviews) reporting guideline. At the identification stage, an information specialist performed a comprehensive search of 6 electronic databases from their inception to May 2021. The inclusion criteria were: all populations; all AI interventions that were used to facilitate SDM, and if the AI intervention was not used for the decision-making point in SDM, it was excluded; any outcome related to patients, health care providers, or health care systems; studies in any health care setting, only studies published in the English language, and all study types. Overall, 2 reviewers independently performed the study selection process and extracted data. Any disagreements were resolved by a third reviewer. A descriptive analysis was performed.

**Results:**

The search process yielded 1445 records. After removing duplicates, 894 documents were screened, and 6 peer-reviewed publications met our inclusion criteria. Overall, 2 of them were conducted in North America, 2 in Europe, 1 in Australia, and 1 in Asia. Most articles were published after 2017. Overall, 3 articles focused on primary care, and 3 articles focused on secondary care. All studies used machine learning methods. Moreover, 3 articles included health care providers in the validation stage of the AI intervention, and 1 article included both health care providers and patients in clinical validation, but none of the articles included health care providers or patients in the design and development of the AI intervention. All used AI to support SDM by providing clinical recommendations or predictions.

**Conclusions:**

Evidence of the use of AI in SDM is in its infancy. We found AI supporting SDM in similar ways across the included articles. We observed a lack of emphasis on patients’ values and preferences, as well as poor reporting of AI interventions, resulting in a lack of clarity about different aspects. Little effort was made to address the topics of explainability of AI interventions and to include end-users in the design and development of the interventions. Further efforts are required to strengthen and standardize the use of AI in different steps of SDM and to evaluate its impact on various decisions, populations, and settings.

## Introduction

### Shared Decision Making

Shared decision making (SDM) is the process in which patients and health care providers collaborate to make decisions based on the latest medical evidence and patients’ preferences and values [1]. In this process, health care providers present the patient with options for screening or treatment and evidence for each option’s harms and benefits. The patient is invited and supported in expressing their preferences and values, and eventually, patients and their health care providers collaboratively make a decision that is best aligned with patients’ preferences and values [1]. Thus, the final shared decision is informed by the best evidence and by what matters most to the patient [2]. The use of SDM in clinical practice has been limited [3-5]. The most frequently reported reasons by health care providers are time pressure, lack of applicability because of patient characteristics, and clinical situations [6].

Elwyn et al [7,8] presented a 3-step model for clinical practice, consisting of team talk, option talk, and decision talk. During team talk, the need to provide support to patients when choices are presented and to elicit their goals to guide decision-making is emphasized. Option talk consists of providing more information about these options and comparing them through risk communication. Finally, during decision talk, health care providers guide patients to a decision based on their experience and expertise, which reflects the informed preferences of patients. The model aims to simplify the process so that health care providers can integrate SDM and patient decision support into their practice. Despite this, the use of SDM in clinical practice faces barriers that can potentially be alleviated by using artificial intelligence (AI).

### Artificial Intelligence and Its Potential in Health Care

AI, defined as “computational intelligence” or the “science and engineering of making intelligent machines” [9], describes the fast-growing field of simulating intelligent, human-like behavior in computers and technology [10]. AI can provide decisional support to health care providers and patients. Machine learning, a subfield of AI, enables computers to learn from data without explicit programming [11,12]. Computers are provided with large data sets and learn to make accurate predictions, for example, on the diagnosis and prognosis of health outcomes of an individual, in different settings, including primary health care [13], identifying patterns and trends and learning from previous experience [14].

In the last 2 decades, AI has been applied in various fields, such as telecommunications [15], financial services [16], and health care [17]. AI has shown promising results in various fields, including radiology [18], ophthalmology [19], cardiology [20], orthopedics [21], and pathology [22]. For example, in medical imaging, AI can be used to assess x-rays, thus reducing the workload of health care providers [23]. It also has the potential to help health care providers assess patients’ health risks, increase the efficiency and effectiveness of intervention and treatment, empower patients to better understand their health and self-manage their conditions, reduce waiting times and costs, and ultimately improve the quality of care and patient outcomes [24-26].

AI has the potential to foster SDM by informing decision-making and allowing health care providers to focus their energy on spending more time with the patient [27]. AI tools provide a wide variety of information with the ability to analyze large amounts of data and discover correlations that may have been missed by researchers and health care providers [28]. There is emerging literature regarding the bioethics and obstacles behind using AI for health decision-making [27], patients’ and health care providers’ perceptions of AI-based decision aids [29] and how it should be incorporated to ensure that health care is patient-centered. However, little is known about how AI is used in SDM in practice and how it can facilitate the decision-making step of SDM. Therefore, we aimed to systematically examine the evidence on the use of AI in SDM through a scoping review to map existing knowledge.

### Objective and Research Question

The objective of the scoping review is to examine evidence on the use of AI in SDM, namely, to explore what has already been done and what future roles may exist for the use of AI in SDM.

Our specific research questions are as follows: (1) What is the available knowledge on the use of AI interventions for SDM? (2) How is AI being used for the decision-making point of SDM?

## Methods

### Study Design

The scoping review methodological framework proposed by Levac et al [30], modifications to the original framework of a scoping review [31], and the Joanna Briggs Institute methodological guidance for scoping reviews [32] were used to guide this research. We developed a protocol with the following steps: (1) identifying the research question; (2) identifying relevant studies; (3) selecting studies using an iterative team approach to study selection and data extraction; (4) charting the data by incorporating a numerical summary; (5) collating, summarizing, and reporting the results; and (6) consulting the results regularly. This protocol is registered and available on the Open Science Framework website [33]. We completed this review according to the published protocol. Finally, we used the PRISMA-ScR (Preferred Reporting Items for Systematic Reviews and Meta-Analyses extension for Scoping Reviews) checklist for reporting [34]. The filled PRISMA-ScR checklist is available in Multimedia Appendix 1.

### Eligibility Criteria

We defined the eligibility criteria for our search using the *Population, Intervention, Comparison, Outcomes, Setting and study designs* components [35].

#### Population

Any population that provided health care (eg, general practitioners, nurses, social workers, pharmacists, and public health practitioners) and any individual who received care (eg, patients and their families and caregivers) were included.

#### Intervention

Any AI intervention implemented or tested during an SDM process in a clinical context was included in the study. AI was defined according to the definition provided by McCarthy [9] and Russell et al [36]. AI interventions included expert systems, knowledge representation, machine learning involving predictive models, reinforcement learning, natural language processing, and computer vision. If the AI intervention was not used for the decision-making point in SDM, it was excluded. We defined SDM as a process that occurred if the following three steps had taken place: (1) team talk, (2) option talk, and (3) decision talk [7,8].

#### Comparators or Control

No limitation.

#### Outcome

Any outcome related to patients, health care providers, or health care systems were included in this study.

#### Setting and Study Design

Studies in any health care setting (eg, primary care and secondary care); all studies using qualitative, quantitative, and mixed methods designs; and only studies published in the English language were included. Reviews, opinion pieces, editorials, comments, news articles, letters, and conference abstracts were excluded.

### Information Sources and Search Strategy

A comprehensive literature search was designed and conducted by an experienced information specialist in consultation with the research team. The seed articles were identified by experts on the team, and the final search strategy was reviewed by the lead author. The process of the literature search was iterative. The following six electronic databases were searched from their inception to May 2021: MEDLINE (Ovid), EMBASE (Ovid), Web of Science Core Collection, CINAHL, Cochrane Library (CENTRAL), and IEEE Xplore Digital Library. The reference lists of the included studies were searched manually. Retrieved records were managed with EndNote X9.2 (Clarivate) and imported into the DistillerSR review software (Evidence Partners) to facilitate the selection process. The final search strategies and key terms for each database are available upon request.

### Study Selection Process

We removed duplicates and then applied the inclusion criteria for level 1 (title and abstract) and level 2 (full text) screening using a standardized inclusion criteria grid. A pilot test of 55 studies (12% of the total 458 citations) for level 1 screening was conducted. Once familiar with the literature of interest, we modified the a priori eligibility criteria to adjust our study selection where necessary. Subsequently, 2 reviewers (PG, MC, and YH) independently screened the titles and abstracts. The reasons for exclusion were recorded for full-text selection. Any disagreements regarding study inclusion were resolved by a third reviewer (SAR).

### Data Items and Data Collection Process

A data extraction form was drafted and finalized with feedback from the team members. Elements for data extraction included study characteristics (eg, year published, country of the corresponding author, and study setting), characteristics of the AI intervention (eg, purpose of the intervention, methods/techniques used, data sources, and performance), involvement of end users in the development of the intervention (eg, health care providers and patients), aspects of the AI intervention (eg, explainability of AI and reproducibility of intervention), whether AI was implemented or tested, how the AI intervention was used for decision-making in SDM, and outcomes (eg, related to patients, health care providers, and health care systems). A total of 2 reviewers (YH, PG, and MC) independently extracted relevant data from each included study. All data were verified by a third reviewer (SAR).

### Critical Appraisal

In alignment with the proposed framework for methodological guidance in scoping reviews, we did not conduct a quality appraisal. Critical appraisal in scoping reviews is not considered mandatory [30-32].

### Synthesis

We summarized our findings using descriptive statistics and performed a narrative synthesis describing the characteristics of the AI intervention, whether end users were involved in the development and/or its validation, how the AI intervention supported the decision point of SDM, and what the outcomes were if it was implemented in a clinical setting. We informed our synthesis through the work and toolkits published by Popay et al [37], titled “Guidance on the conduct of narrative synthesis in systematic reviews.” Specifically, we performed a thematic analysis and identified 3 main themes across the included studies in an inductive manner (involvement of end users, outcomes of AI interventions, and AI interventions for the decision point). This allowed us to organize and present our results comprehensively.

### Consultation

The results were provided to the team members for their feedback. Study updates were also provided to the researchers and health care providers during 2 workshops led by the first author (SAR) at 2 international scientific conferences, that is, the 10th International Shared Decision Making Conference and the annual meeting of the North American Primary Care Research Group.

## Results

### Flow of Studies

**Figure 1:**
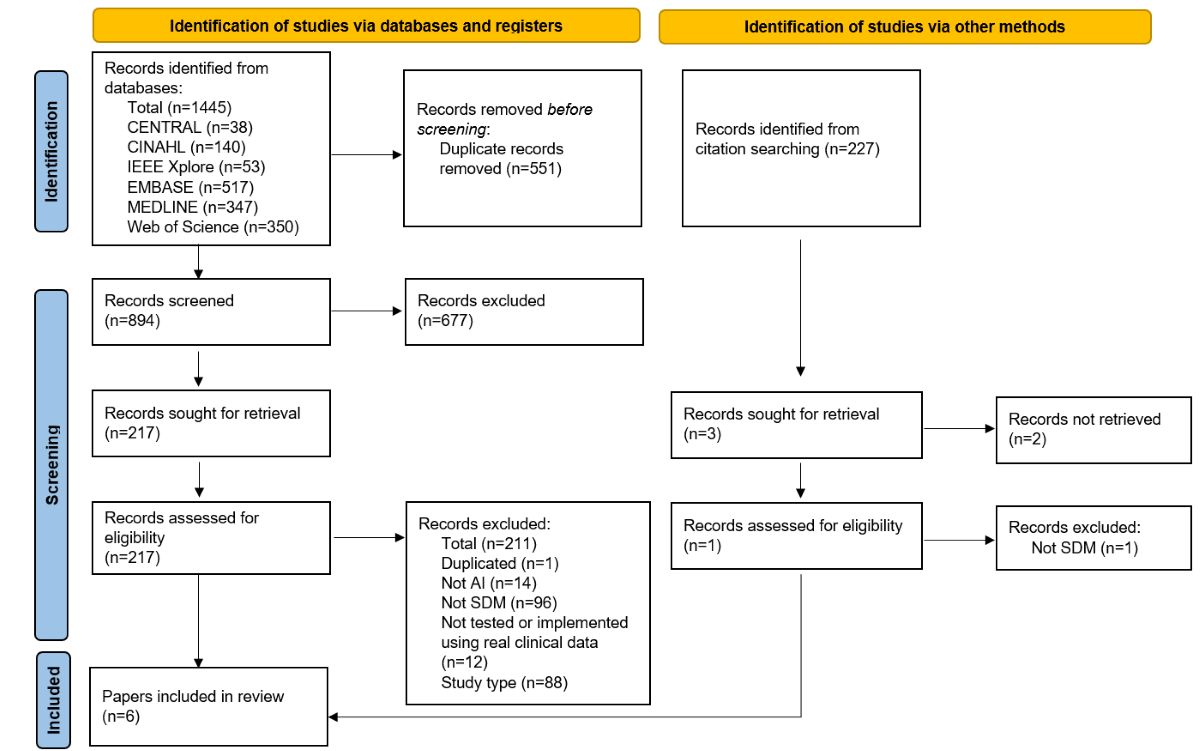
PRISMA (Preferred Reporting Items for Systematic Reviews and Meta-Analyses) flow diagram. Adapted from Page et al [40]. AI: artificial intelligence; SDM: shared decision making.

The search process resulted in 1445 records from the selected electronic databases, 551 of which were excluded as duplicates. Of the remaining 894 studies, we excluded 677 at level 1 screening because they did not meet the inclusion criteria and the remaining 217 underwent full-text review. Citations were manually searched (n=227), of which 3 studies were sought for retrieval and was assessed for eligibility. No eligible studies were found in the reference search. Ultimately, 6 articles met our inclusion criteria (Figure 1). Of 6 articles, 2 referred to the same study [38,39]. The full list of included articles and their details can be found in Multimedia Appendix 2 [34-39].

### Characteristics of Included Articles

**Figure 2:**
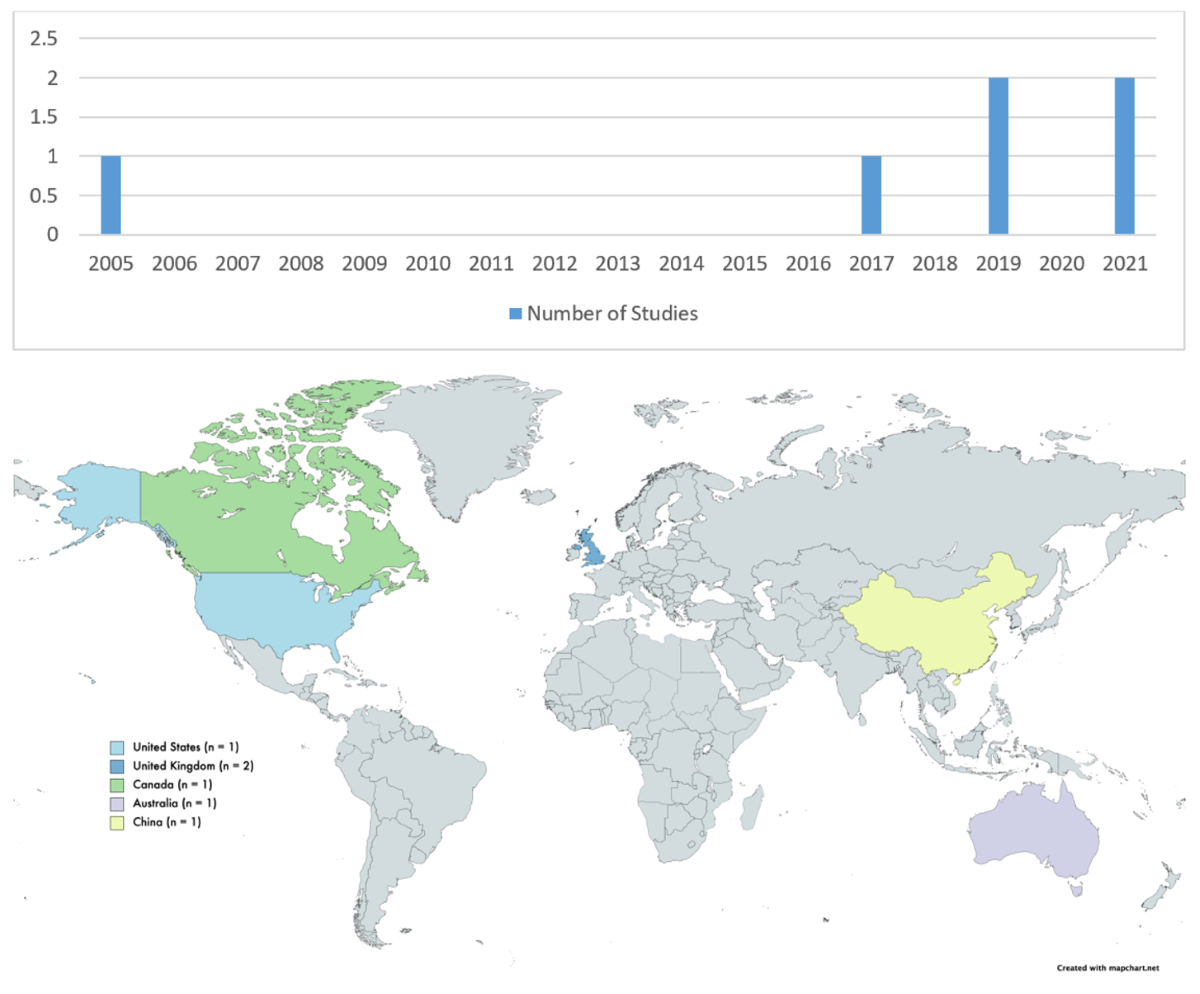
Years of publication and countries where studies are outlined in the included papers.

The number of studies published annually has increased since 2017, with the majority conducted in North America and Europe. The distribution and publication dates of the included studies are shown in Figure 2.

### AI Characteristics—Purpose, Development, Data Sets, and Performance

In Table 1, we highlight the AI characteristics of the included studies, such as the AI method used, characteristics of the data set, and performance measures.

**Table 1:** Characteristics of artificial intelligence (AI) interventions.

Study	AI method	Data set and its characteristics	Performance
Frize et al [41]	Machine learning, artificial neural networks, and case-based reasoning	Not provided	Not provided
Wang et al [42]	Machine learning, multilabel classification methods, k-nearest neighbors, and random k-label sets	Electronic health records 2542 patients 65.6% male, 34.4% female Mean age 66.46 (SD 13.81) years 70% of this was used for training, and 30% was used for testing	Performance accuracy of 0.76
Twiggs et al [43]	Machine learning, Bayesian belief network, and Bayes network	Data from the National Institutes of Health Osteoarthritis Initiative 330 patients, between the ages of 45 and 79 years, have undergone total knee arthroplasty	Not provided
Jayakumar et al [44]	Machine learning (type not specified)	Not provided	Not provided
Kökciyan et al^a^ [38,39]	Metalevel argumentation frameworks	Not provided	Not provided

^a^This refers to both articles describing the system developed by Kökciyan et al [38,39], which were included.

Of the included articles, all used machine learning as the type of AI. Only 2 articles presented information on the data set used to develop the AI intervention [42,43], and 1 article reported the performance accuracy (0.76) of their intervention [42].

Most of the included articles (n=4) did not report on the data set used to develop the AI intervention; among those that did (n=2), only 1 reported on the sex distribution of the patient data [42], and both provided information about the age (mean or range) of patients in their data set. Only 1 article specifically mentioned the breakdown of data used to develop and test their intervention [42] but did not report data set characteristics for the 2 breakdowns. None of the included articles commented on the generalizability of the algorithm or representativeness of the data used to develop and train the AI intervention. Although 2 articles mentioned the aspects of explainability and interpretability [39,43], none of the included articles reported on how they developed their AI interventions to be explainable and/or interpretable.

Explainable AI is a broad and new domain and is being studied in AI. In general, we can consider explainability throughout AI development: (1) *premodeling explainability*, (2) *explainable modeling*, and (3) *postmodeling explainability*. One of the challenges in this field is the so-called explainability versus performance trade-off (often, high-performance methods such as deep learning are less explainable).

In health care, explainability and interpretability are required for patients and health care providers to understand why AI interventions produce a certain prediction or suggestion and to trust this output [45]. Without this understanding, ethical and practical challenges arise, including a lack of trust and transparency in AI tools [28]. A lack of explainability and interpretability creates an informational discrepancy between patients and health care providers, impeding risk assessment and giving rise to ethical issues such as the ascription of responsibility when an incorrect decision is made [28]. Moreover, a lack of explainability and interpretability ties into the issue of informed consent in health care [46]. It is unclear as to what level of understanding patients who use AI require to provide informed consent and to what extent health care providers are responsible for educating their patients on its use [46]. However, explainability and interpretability are crucial in increasing the transparency of the inner workings of the system and in fostering the trust of health care providers and patients in the outcomes the AI may provide throughout the process of SDM [45].

Frize et al [41] developed and tested a decision support system that used AI to tailor information to help parents decide to continue, limit, or discontinue intensive care of newborns [41]. Machine learning methods, such as artificial neural networks and case-based reasoning methods, were used in this decision support system. The AI component was capable of knowledge learning, processing, and derivation. The developed system was able to provide structuralized knowledge translation and exchange between all participants and facilitate collaborative decision-making. Overall, clinicians found the classification rates of the model acceptable in comparison with the constant predictor used as a statistical benchmark, but no other performance metrics were presented.

Wang et al [42] proposed an SDM system framework connected to the electronic health records (EHRs) of patients with type 2 diabetes to provide them and their health care providers with tailored knowledge and choices about medications [42]. Machine learning methods, multilabel classification methods including k-nearest neighbors algorithms, and random k-label sets using EHR data were used to provide a medication recommendation list based on patients’ current conditions. The data set used to develop the AI intervention included data from 2542 patients. Of these, 65.62% (1668/2542) were men and 34.38% (874/2542) were women. The mean age of the included patients was 66.46 (SD 13.81) years. Associated diseases and vital sign values were also reported. The authors used 70% of the total data set to train the AI algorithm, and the remaining 30% to test it. The AI model had an accuracy of 0.76.

Twiggs et al [43] developed a clinical tool to predict total knee arthroplasty outcomes for patients with advanced osteoarthritis to help patients and surgeons decide whether a surgical or nonsurgical pathway is most appropriate on a patient-specific basis. The group developed a Bayesian belief network to identify patients at risk of limited improvement from total knee arthroplasty using data from the National Institutes of Health Osteoarthritis Initiative, a publicly accessible database. A total number of 330 patients between the ages of 45 and 79 years who had undergone total knee arthroplasty were included. The team used a machine learning method, that is, a naive Bayes network, for variable selection and model generation.

Jayakumar et al [44] performed a randomized clinical trial to assess whether an AI-based decision aid influenced decision quality, patient experience, functional outcomes, and process-level outcomes in patients with advanced osteoarthritis considering total knee replacement. They used a machine learning–based platform to generate personalized outcomes. Neither the development nor the performance of the model was described in the article; however, they mentioned that the AI intervention had been tested in a clinical setting and that its fidelity had been discussed with the clinical team before deployment.

Kökciyan et al [38,39] developed a decision support system, that is, “CONSULT,” to help patients who had stroke in self-management and adherence to treatment plans, in collaboration with health care providers. Patients, caregivers, and health care providers collaborate to decide the best treatment plan for the patient. The system was developed using metalevel argumentation frameworks. Wellness sensor data, EHR data, and clinical guidelines were used as input, and recommendations and textual explanations for automated decisions were provided as output.

### Involvement of the End Users

In terms of end user (ie, patients and health care providers) involvement in the design, development, and/or validation of AI systems, we found that 3 of the articles [39,41,44] included health care providers to validate the AI intervention, and 1 of the articles included both health care providers and patients in clinical validation of their AI tool [43]. The first 3 articles involved clinicians validating the correctness of the recommendations and explanations provided to patients by the AI tool [39], confirmed the fidelity of the AI intervention before deployment [44], and were included in the testing of usability and acceptability as well as a needs assessment of the intervention [41]. Twiggs et al [43] clinically validated their tool for both patients and health care providers.

One of the articles [38] also held initial patient focus groups in which co-design activities were held. These activities resulted in a user-centered version of how they wished to see the information displayed by the decision support tool. No additional information on how the co-design activities were organized was provided.

### Population Characteristics and Outcomes

In total, 4 of the included articles tested their interventions for usability and acceptability [38,39,41,42], and 2 of the articles implemented their interventions in clinical settings with targeted end users (eg, patients and health care providers) [43,44]. Only the last 2 articles reported outcomes related to patients and health care providers. These were primarily psychosocial outcomes and included better decisional quality, improved SDM, increased satisfaction, and better clinical postoperative outcomes. Of the included articles, 3 also reported outcomes related to health care systems [42-44]. These were related to the general workflow and how the interventions did not significantly alter the flow or time it took to provide care. They also include the high feasibility and convenience of integrating AI into health care systems.

All the included articles provided some level of detail related to the population of the data sets that they used to train or test their algorithm. Only 1 article provided a thorough presentation of the population by reporting the sociodemographic characteristics of the participants involved [44]. In total, 4 articles tested the interventions for usability and acceptability, whereas 2 articles observed actual outcomes by applying their intervention in clinical contexts [43,44].

Frize et al [41] tested their AI for acceptability and usability with an expert panel consisting of a neonatologist, engineer or computer scientist, clinical nurse specialist, social worker, and ethicist. The classification rate of the intervention was found to be acceptable for a clinical trial tool. The needs assessment performed through interviews with 5 neonatal clinicians confirmed that the design of their tool met the needs of the population for which it was designed. Acceptability was evaluated using open-ended questions based on a questionnaire from the Foundation for Informed Medical Decision-Making. The expert panel found the tool clear and easy to use.

Kökciyan et al [38,39] performed a pilot study using their CONSULT system to assess its usability and acceptability. The system was implemented as a mobile Android app, and 6 healthy volunteers were recruited to use the system for a week. They interacted with different aspects of the system and were asked to regularly collect measurements from wellness sensors and input data. A pilot study demonstrated the usability of the app.

Wang et al [42] tested their AI interventions using clinical data. The authors used 30% of the clinical data set mentioned earlier to test the AI intervention. The total data set included data on 2542 patients, of which 65.6% (n=1668) were male. As these EHRs only included hospitalized patients, the outcome of medication use was not considered. In terms of outcomes for health care systems, the intervention was reported to have high feasibility and maintenance—if the model or knowledge required for proper function became outdated, the intervention could be modified without affecting the normal operation of the hospital’s EHR system.

Jayakumar et al [44] conducted a randomized clinical trial that recruited 129 patients with presumptive knee osteoarthritis who were candidates for primary total knee replacement. A total of 69 patients were in the intervention group (n=46, 67% women) and 60 were in the control group (n=37, 62% women). The mean age of the intervention group was 62.59 (SD 8.85) years, whereas the mean age of the control group was 62.62 (SD 7.81) years. The authors reported on ethnicity, education, work status, social status, and insurance status for both the intervention and control groups. The control group received an educational module and usual care, whereas the intervention group received a preference model and an output from the AI tool. Both groups met the surgeons afterward for the decision-making discussion. In terms of patient-related outcomes, the intervention group showed better decisional quality and improved SDM, patient satisfaction, and functional outcomes. Overall, the use of the AI tool did not prolong consultation times.

Twiggs et al [43] performed a clinical validation with 150 patients who presented to a surgeon with >30 years of experience in 2 cohorts. They included patients aged ≥55 years with knee pain without a history of meniscal or ligamentous injury. They collected data over 3 months. Patients were first asked to fill a digital questionnaire based on knee osteoarthritis and injury outcome scores, as well as demographic and medical condition data. These data were used by their developed intervention to calculate a predictive postoperative score and display it visually on a percentile scale of the pain of a population of patients with osteoarthritis seeing a surgeon. The first cohort consisted of 75 (50%) consenting patients who filled the group’s developed questionnaire. In this cohort, the surgeon and patients were blinded to the predictive output of the tool and proceeded with their consultations as normal. The second cohort consisted of 75 (50%) consenting patients, and both the patients and surgeons were exposed to the output of the intervention. The outcomes were reported for patients and surgeons. Although the use of the AI intervention output did not change the proportion of patients booked for total knee arthroplasty surgery, there was a change in the level of patient-reported pain between those booked and not booked for surgery when using the tool. Apart from the questionnaire, which only took 10 minutes to complete, there was no disruption to the normal surgeon consultation workflow.

### AI Interventions for the Decision Point

Of the included articles, 3 designed AI interventions for primary care [38,39,42], relating to the care of individuals with chronic conditions including patients with diabetes and stroke survivors, and 3 for secondary care [41,43,44], of which 2 (67%) focused on patients requiring treatment for their knee and 1 (33%) focused on neonatal intensive care. The included articles supported the decision-making step of SDM by introducing interventions to predict outcomes [41,43,44] of clinical significance and for clinical recommendations [38,39,42]. In Table 2, we provided information about the setting, decision-making problem, and a summary of how AI is being used for decision-making in SDM.

**Table 2:** Summary of artificial intelligence interventions and how they are being used for decision-making in the included studies.

Study	Setting	Decision-making problem	AI^a^ for decision-making
Wang et al [42]	Primary care	Knowledge and choices about antihyperglycemic medications	The tool provides patients and health care providers with tailored knowledge and choices about antihyperglycemic medications through the integration of electronic health record data. Patients and physicians can review patients’ conditions more comprehensively and tailor consultations to the patient’s current condition.
Frize et al [41]	Secondary care	Neonatal intensive care decisions	The tool allows health care providers to predict outcomes in neonatal intensive care and counsel families on the pros and cons of deciding to initiate or withdraw treatment. The tool also promotes parental involvement in the decision-making process.
Twiggs et al [43]	Secondary care	The decision about total knee arthroplasty	The AI intervention presents end users (patients and surgeons) with interpretable information relating to the risk of no improvement after total knee arthroplasty. This helps them decide whether to proceed with total knee arthroplasty.
Jayakumar et al [44]	Secondary care	The decision about total knee replacement	AI system provides patients with a personalized outcome report, which is then discussed with the surgeon during decision-making discussions.
Kökciyan et al [38,39]^b^	Primary care	The decision about treatment plans and options for stroke survivors	This tool supports the decision-making point by providing an up-to-date view of the patients’ situation based on personalized metrics and provides explanations for its recommendations.

^a^AI: artificial intelligence.

^b^This refers to both articles describing the system developed by Kökciyan et al [38,39] that were included.

The AI intervention by Wang et al [42] supports the decision point by providing patients and health care providers with tailored knowledge and choices about antihyperglycemic medications through the integration of EHR data. Their tool was designed with specific end-user interfaces for each step of SDM (team talk, option talk, and decision talk). During decision talk, patients can have more efficient conversations with their health care providers based on the medication recommendations that the AI system provides. It is designed for both inpatient and outpatient settings and provides a more intuitive understanding of patient conditions and knowledge of diabetes medications.

The AI intervention by Frize et al [41] supports the decision point as the components of the tool interact to provide predictive analysis, document repository, customized delivery, and adaptive interfaces. They aimed to augment group clinical processes in various phases of decision-making. The goal was to promote parental involvement and collaboration with the clinical team. The tool allows health care providers to predict outcomes in neonatal intensive care and counsel families on the pros and cons of deciding to initiate or withdraw treatment.

The tool presented by Twiggs et al [43] supports the decision point by presenting end users, that is, patients and surgeons, with interpretable information relating to the risk of no improvement following total knee arthroplasty. It provides interpretable output, allowing end users to understand the impact of alternative treatments. This tool helps patients and their surgeons decide whether they are good candidates for the procedure.

The intervention by Jayakumar et al [44] supports the decision point by providing patients with a personalized outcome report based on data inputs (ie, demographics, patient-reported outcome measurements, and clinical comorbidities), which is discussed with the surgeon during the decision-making.

The CONSULT system by Kökciyan et al [38,39] supports the decision-making point in SDM by presenting an up-to-date view of the patient’s situation based on personalized metrics, from a patient’s EHR and wireless sensor input and providing textual explanations of automated decisions of the tool to accompany the recommendations it provides. The relevant, up-to-date, summarized data CONSULT provides, along with treatments and recommendations, support the decision-making point between patients and their health care professionals.

## Discussion

### Principal Findings

We conducted a scoping review as a first step toward a comprehensive overview of the literature on the use of AI in SDM. This overview provides a basis for future systematic review. The results of our study lead us to make the following observations.

### Role of AI in SDM

The included articles presented AI interventions used for decision-making during SDM in similar ways. Within the included articles, AI interventions were specifically applied to predict outcomes of clinical significance and for clinical recommendations. The decision-making step can benefit from AI interventions because AI can present a comprehensive and personalized list of treatment options, as well as risks and benefits, thus increasing the amount of knowledge related to the condition, treatment, side effects, risks, and outcomes. AI models are capable of learning and processing all information related to a patient’s care and can generate evidence-based recommendations to support SDM [47]. These models can also be used to support risk communication. Similar to how they may be integrated into an intelligent tutoring system, predictive models can present relevant information when discussing risks associated with a patient’s condition in a manner appropriate for that specific patient, as well as assess their level of understanding and provide supplementary information accordingly [48].

The decision-making step is a core step of SDM, in which patient–health care provider interaction is essential and should remain independent of and unrestrained by AI intervention. Patient–health care provider relationships are based on responsibilities that provide a foundation for the relationship to grow. Despite acknowledging the benefits AI may have on facilitating SDM, patients continue to expect their health care provider to retain final discretion over treatment plans and monitor their care, as well as to adapt any contribution from the AI intervention to their unique situation [49]. Conversely, patients expect to remain empowered in decision-making and can either dispute or refuse the input of AI [49]. It is important to design and implement AI interventions in clinical settings in a way that does not negatively impact the human and personal aspects of certain decisions during the SDM process. AI interventions must be implemented in ways that preserve and uplift patient–health care provider relationships in care, as well as facilitate making shared medical decisions.

AI interventions can open up more time for health care providers to spend connecting with their patients; however, they may place the health care provider in a mediator-like role, in which they will be responsible for explaining the AI output to their patients. This can be difficult to achieve, especially when a lack of interpretability and explainability may exist in certain AI models, such as deep learning. This lack of interpretability and explainability can result in a lack of trust and decisional delay or conflict consequently, which are factors that SDM aims to resolve [27]. AI interventions in health care can influence patient–health care provider relationships [27], but little is known about how they influence this relationship and what are the best ways to integrate AI into SDM, to use its benefits and mitigate potential risks. Further work is required to investigate how the different steps of SDM can benefit from AI intervention without affecting the patient–health care provider relationship.

### Explainability and Interpretability of AI Systems

One of the principal challenges in the incorporation of modern AI interventions into health care is explainability and interpretability. This refers to the insight an AI intervention gives to clarify its function to an audience; that is, *how* an algorithm generates output from a given input [50-52]. The levels of explainability and interpretability depend on the AI method used. This is the case in certain AI models such as deep learning.

Despite the promising performance of AI, its implementation in clinical practice remains challenging. Trust in AI is one of the main barriers to its adoption in clinical practice [53]. The inability of humans to understand why an AI system makes particular decisions limits the effectiveness of the new generation of AI systems in critical settings, such as primary health care. Prior work has highlighted the significance of explainable AI in health care and has shown that the lack of explainability (*black box*) in AI systems can affect physicians’ and patients’ trust in AI [54-56].

In our review, 2 of the included articles [39,43] briefly touched on explainability and interpretability, stating that textual explanations were provided by the AI tool to explain automated decisions [39] and that the outcome of their AI model is interpretable [43]. However, these 2 articles did not explain the steps they had taken in the development of their tool to make it explainable or interpretable, and none of the other included articles considered these aspects. This might introduce barriers to the implementation of these systems in the process of SDM in clinical practice. As in any other context that attempts to integrate AI into sensitive human interactions, AI explainability, and interpretability for SDM needs to be addressed.

Moreover, the level of understanding of the explainability and interpretability of AI tools might differ for various stakeholders. For instance, an AI expert trained in this field can understand and interpret the reasoning behind an AI algorithm better and quicker than a nonexpert in AI. Therefore, health care providers and patient education about AI can lead to a better understanding of the algorithm, which leads to a better understanding of the explainability of an AI intervention. In brief, end users’ understanding of the predictions/decisions made by the AI intervention, as well as increased explainability and interpretability of the AI tool, can increase end-user *trust* in the outcome given [57].

A lack of trustworthiness is one of the many bioethical barriers that may arise when implementing an AI intervention in health care and SDM; therefore, improving AI literacy in both patients and health care providers, as well as increasing the explainability and interpretability of AI systems, trust can be increased. In addition, there is a discrepancy in the literature regarding the level of explainability required within the health care setting to ensure a proper understanding of and trust in the outcomes provided by the algorithm [58]. Future studies are required to determine how to efficiently educate end users about AI-SDM tools, how to efficiently incorporate explainability and interpretability in this context, and how much explainability and interpretability are deemed sufficient in this context and the context of informed consent.

### Human-Centered AI

Of the included articles, 3 [39,41,44] involved health care providers in the validation stage of the AI system, and 1 included both health care providers and patients in the clinical validation stage of the AI system [43]. One article [38] included patients and health care providers in co-design activities, resulting in user-generated versions of the developed tool. However, no details were provided on how the co-design activity was organized, and end users were not involved in the subsequent design and development of the AI tool.

Further efforts are needed, both from the AI and SDM communities, to include health care providers and patients (as end users of the developed AI systems) in the design, development, validation, and implementation of AI-SDM tools. SDM is the core of patient-centered care; thus, patient values and preferences need to be considered in every step defining the process. Ethicists argue that by not using patient preferences or values as input or influencing the output, but rather leaving the *shared decision* aspect to the patient choosing from evidence-based options presented by the AI, the process is not truly patient centered [59].

Thus, to ensure that SDM fundamentally occurs when AI interventions are introduced, patient preferences must be incorporated into the design. Termed *value-sensitive design*, this method incorporates human values throughout the design process [59]. However, the successful incorporation of individual patient values into algorithm design and how to efficiently include patients and health care providers in the development and validation of AI systems in health for SDM remains a challenge, and further studies are required. A recent assessment of the current methods showed that most existing user-centered design methods were primarily created for non-AI systems and did not effectively address the unique issues in AI systems [60]. This is also the case for AI-SDM tools.

### Reporting on AI Interventions

In our review, we observed poor reporting of AI interventions in the included studies. Studies that report AI interventions should use validated frameworks and guidelines to report their results. Transparent and complete reporting of AI interventions supporting SDM is important for detecting errors and potential biases and evaluating the usefulness of the intervention [61]. An example of such a reporting framework is the Transparent Reporting of a multivariable prediction model of Individual Prognosis or Diagnosis (TRIPOD), which consists of a checklist of items deemed essential for transparent reporting [62]. As the original framework is primarily applied to regression-based predictive models, the TRIPOD-AI extension is being developed, specifically for machine learning–based prediction model studies [63]. Transparent and complete reporting allows for a good understanding and encourages reproducibility of the work in future studies, which is an important factor to consider in the growing implementation of AI-SDM in clinical settings.

None of the articles included in this review mentioned adhering to a specific reporting framework or considered reproducibility. This resulted in a lack of clarity in the included articles regarding different aspects, including whether the training data set was representative, how the potential bias (eg, representativeness and algorithmic biases) and missing data were considered, how AI had been used in the clinical setting, and what were the outcomes resulting from AI implementation. In fact, only 1 article [44] comprehensively reported on the sociodemographic characteristics of the participants involved in the use of AI intervention. Such reporting should be standardized so that AI interventions and clinical implementations can be better understood and compared effectively. The importance of using a reporting framework needs to be emphasized in future AI studies to promote an increased understanding and reproducibility of AI-SDM in clinical contexts.

### Limitations of the Study

We did not conduct a quality appraisal of the included articles, although it is not common, nor is it required to include within a scoping review. However, our review sheds light on this important area, and there are some areas for improvement. Our inclusion criteria were quite strict, and only included articles in which AI intervention was used to support the decision-making point in SDM. Therefore, we may have missed work related to other aspects of SDM. Further systematic reviews may be needed in this area to ensure that the results of this review can be applied in policy and practice.

### Conclusions

In this scoping review, we demonstrated the extent and variety of AI systems being tested and implemented in SDM, showed that this field is expanding, and highlighted that knowledge gaps remain and should be prioritized in future studies. Our findings suggest that existing evidence on the use of AI to support SDM is in its infancy. The low number of included studies shows that not much research has been conducted to test, implement, and evaluate the impact of AI on SDM. Future research is required to strengthen and standardize the use of AI intervention in different steps of SDM and to evaluate its impact on particular decisions, populations, and settings. Greater focus and effort from the research community needs to be made on addressing the aspects of explainability, interpretability, reproducibility, and human-centered AI, especially when developing an intervention of their own. Finally, future research should further investigate which SDM steps will benefit most from what type of AI and how AI interventions can be applied to enforce the patient–health care provider relationship.

## Appendix

Multimedia Appendix 1 PRISMA-ScR (Preferred Reporting Items for Systematic Reviews and Meta-Analyses extension for Scoping Reviews) checklist containing the page number where each reporting criterion is addressed.

Multimedia Appendix 2 Detailed data extraction table.

## Abbreviations

AI: artificial intelligence
EHR: electronic health record
PRISMA-ScR: Preferred Reporting Items for Systematic reviews and Meta-Analysis extension for Scoping Reviews
SDM: shared decision making
TRIPOD: Transparent Reporting of a multivariable prediction model for Individual Prognosis or Diagnosis
