# Supplementary material for: Application of Artificial Intelligence in Shared Decision Making: Scoping Review
Source: JMIR Med Inform. 2022 Aug 9;10(8):e36199. doi: 10.2196/36199 (PMC9399841; doi:10.2196/36199)
Supplement: Multimedia Appendix 2 [file medinform_v10i8e36199_app2.docx]

**Multimedia Appendix 2:** Detailed data extraction table.

| **Study Characteristics** | | | | | **AI Intervention Characteristics** | | | | **Involvement of End-Users** | | **Aspects of AI Intervention** | | **Testing/Implementation and Outcomes** | |
| --- | --- | --- | --- | --- | --- | --- | --- | --- | --- | --- | --- | --- | --- | --- |
| Report Title | First Author | Country | Year published | Study Setting | Clinical Context AI Tool Developed For | Method/Technique of AI | Data Sources | Performance/Accuracy | Involvement of Healthcare Providers in Development | Involvement of Patients in Development | Explainability of AI Intervention | Reproducibility of AI Intervention | Tested/Implemented and Outcome (Related to Patients, Healthcare Providers, Healthcare Systems) | Population Involved in Testing/Implementation |
| Conceptual Framework of Knowledge Management for Ethical Decision-Making Support in Neonatal Intensive Care | Frize, Monique | Canada | 2005 | Secondary care | Neonatal Intensive Care | Machine Learning (ANN-Artificial Neural Networks) + Integrated Knowledgebase Repository | A. EBEs and Input Data Records B. Physician Perspective Data C. Parents Perspective Data  Nothing mentioned about Database Characteristics, which year and where. | Not mentioned | This paper used an expert panel composed of a neonatologist, engineer/computer scientist, clinical nurse specialist, social worker, and ethicist to review and validate the acceptability and usability of the graphical interface of the user interface of their tool. | No | Not mentioned | Not mentioned | Tested   The tool was found easy to use, but no outcomes related to patients, healthcare providers or healthcare systems were reported. | An expert panel composed of a neonatologist, engineer/computer scientist, clinical nurse specialist, social worker, and ethicist |
| A Shared Decision-Making System for Diabetes Medication Choice Utilizing Electronic Health Record Data | Wang, Yu | China | 2017 | Primary Care | Diabetes Care in a Primary Care Center | Machine learning  (Multilabel classification method including k-nearest neighbours algorithms [kNNs] and random k-labelsets) | Electronic health records (EHRs)  2542 EHRs Total 70% used for testing 30% used for training | 0.7611 | No | No | Not mentioned | Not mentioned | Tested | Of the total 2542 EHRs, 1,668 (65,6%) were male and 874 (34.4%) were female. The mean age of the included patients was 66.46 ±13.81 years. Concomitant diseases and vital sign values were also reported. |
| Clinical and Statistical Validation of a Probabilistic Prediction Tool of Total Knee Arthroplasty Outcome | Twiggs, Joshua | Australia | 2019 | Secondary care | Total knee arhtroplasty (TKA) outcomes in patients with advanced osteoarthritis | Bayesian belief network (BBN) and Bayes network | A publicly accessible database created and maintained by the National Institutes of Health Osteoarthritis Initiative (OAI), 4796 volunteers aged 45-79 years over the course of 108 months. The BBN was developed using all patients who had undergone a TKA within the dataset, totaling 330 at the time of development. For each of the 330 patients, a pool of 110 potential preoperative variables were identified. | A Spearman’s rho correlation was found to be 0.53 giving an R2 of 0.29. | No | No | No, although mention that the outcome of their AI model is interpretable. | Not mentioned | Implemented  The use of the AI intervention output did not change the proportion of patients booked for total knee arthroplasty surgery. There was a change in the level of patient-reported pain between those booked and not booked for surgery, when using the tool. Apart from the questionnaire which only took 10 minutes to fill out, there was no disruption to normal surgeon consultation workflow. | 1 surgeon with over 20 years of experience, 150 patients |
| Comparison of an Artificial Intelligence-Enabled Patient Decision Aid vs Educational Material on Decision Quality, Shared Decision-Making, Patient Experience, and Functional Outcomes in Adults With Knee Osteoarthritis: A Randomized Clinical Trial | Jayakumar, Prakash | United States | 2021 | Secondary care | Patients with advanced osteoarthritis considering total knee replacement | Machine learning | Not mentioned | Not mentioned | Although not specifically in development, there was testing within the clinical setting, and discussions about the fidelity of the AI between the clinical team and the company. | No | Not mentioned | Not mentioned | implemented  The intervention group showed better decisional quality, improved shared decision making, patient satisfaction and functional outcomes. Use of the AI tool did not prolong consultation times. | A total of 129 patients referred with presumptive knee OA and candidacy for primary TKR were recruited between March 2019 and January 2020.   69 patients were in the intervention group (67% women) and 60 were in the control group (62% women). The mean age of the intervention group was 62.59, with S.D. of 8.85 years, while the mean age of the control group was 62.62 with S.D. of 7.81. The authors reported on ethnicity, education, work status, social status, and insurance status for both the intervention and control groups as well. |
| A Collaborative Decision Support Tool for Managing Chronic Conditions | Kökciyan, Nadin | United Kingdom | 2019 | Primary care | Stroke survivor self-management and adherence to treatment | Argumentation engine, based on ASPIC+ | Biometric data from wellness sensors from patients, patient EHR data | Not mentioned | No | Co-design activities were included in initial stroke patient focus groups, which resulted in user-generated versions of how information should be displayed. | Not mentioned | Not mentioned | Tested |  |
| Applying Metalevel Argumentation Frameworks to Support Medical Decision Making | Kökciyan, Nadin | United Kingdom | 2021 | Primary care | Stroke survivor self-management and adherence to treatment | Argumentation framework | Biometric data from wellness sensors from patients, patient EHR data | Not mentioned | A healthcare professional in the project team assessed the correctness of the recommenda- tions as well as the explanations provided to the patients. | No | Not specifically; textual explanations are provided by the AI tool to explain automated decisions. | Not mentioned | Tested  The system was implemented as a mobile Android application, and six healthy volunteers were recruited to use the system for a week. They interacted with different aspects of the system, and were asked to collect measurements from wellness sensors and input data on a regular basis. The pilot study demonstrated usability of the application.  Future studies with stroke patients have been approved. | 6 healthy volunteers |
